# Supplementary material for: Integrative Analysis of Angiogenesis-Related Long Non-Coding RNA and Identification of a Six-DEARlncRNA Signature Associated with Prognosis and Therapeutic Response in Esophageal Squamous Cell Carcinoma
Source: Cancers (Basel). 2022 Aug 30;14(17):4195. doi: 10.3390/cancers14174195 (PMC9454540; doi:10.3390/cancers14174195)
Supplement: Supplementary file 1 [file cancers-14-04195-s001.zip › cancers-1840978-supplementary.pdf]

Supplementary Materials

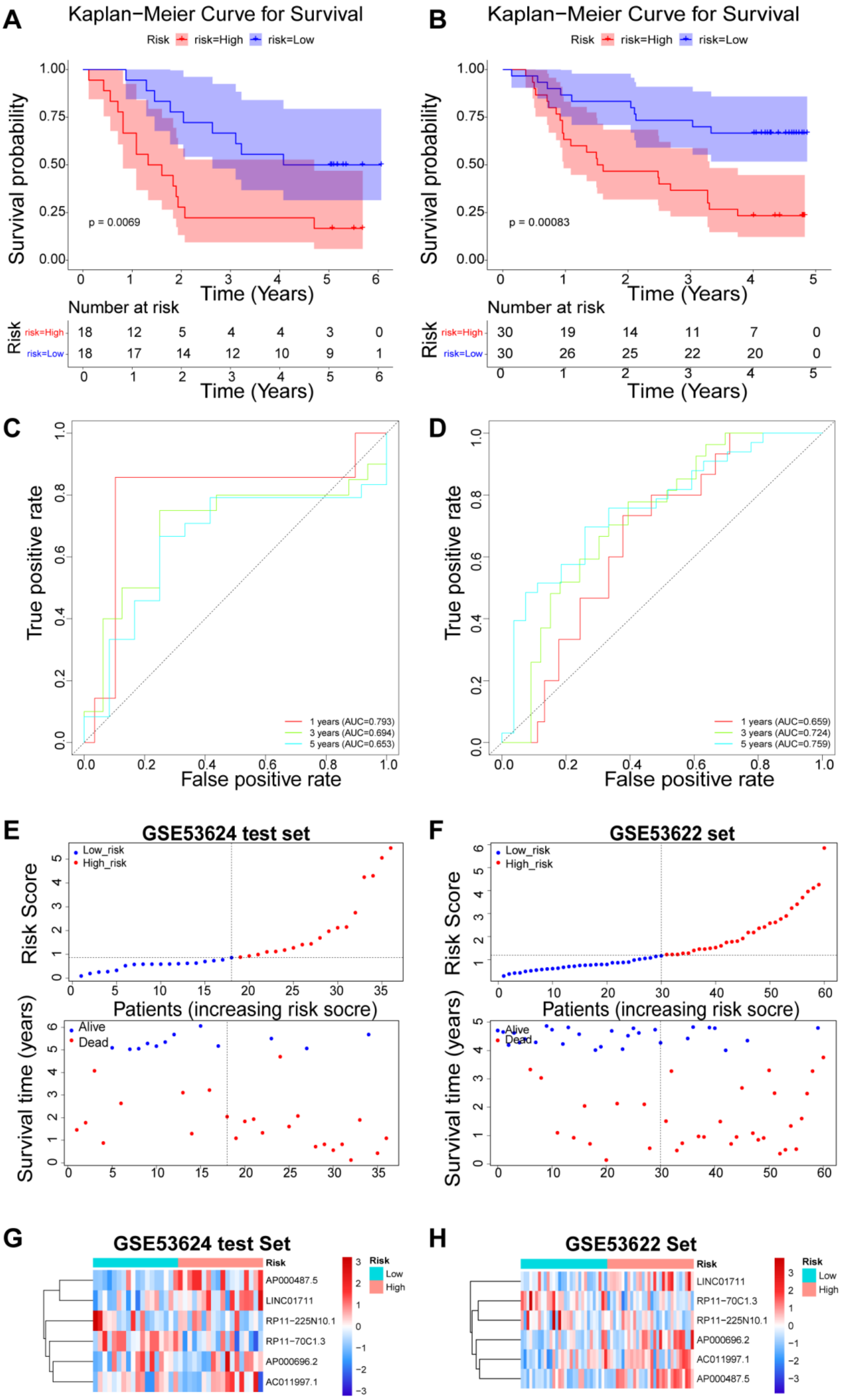

Figure S1. Prognostic values and predictive accuracy of the six-DEARlncRNA. K-M survival curve, Time-dependent ROC curve, Risk score distribution, survival overview, and heatmap for patients in the GSE53624 test set (A,C,E,G) and GSE53622 validation set (B,D,F,H) assigned to high- and low-risk groups based on the risk score.

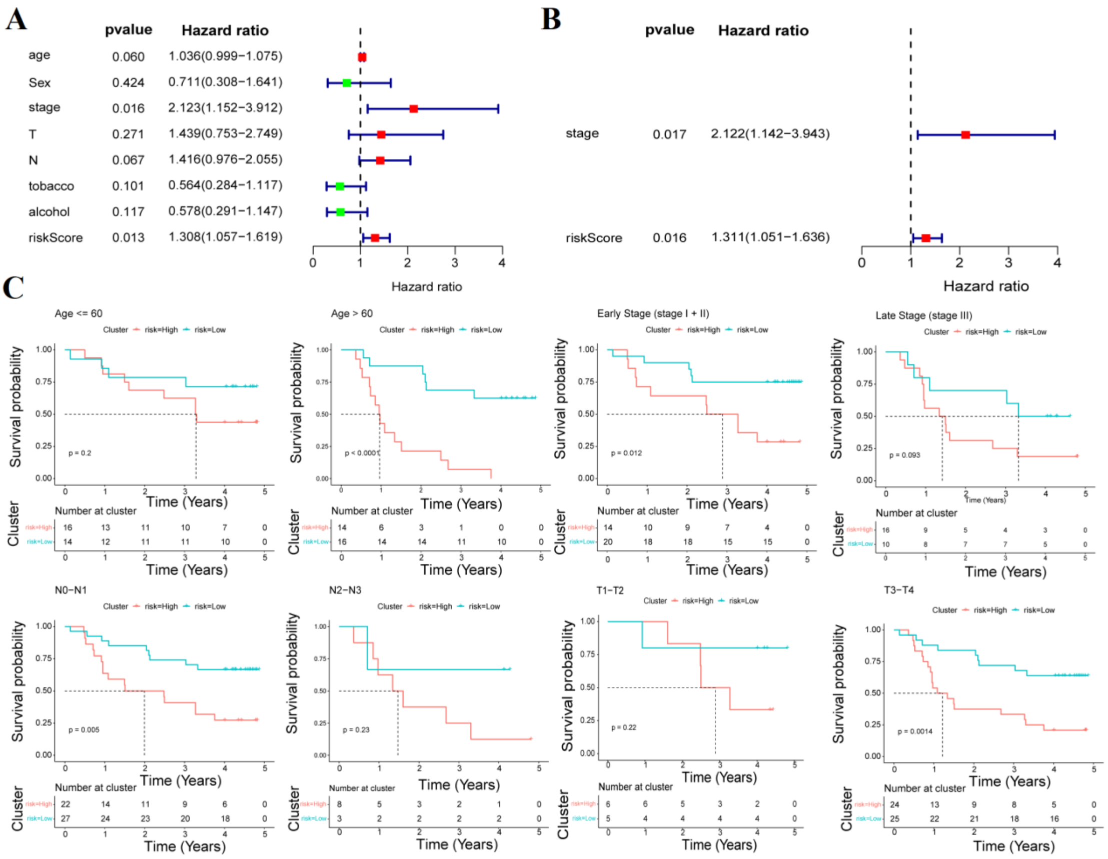

Figure S2. A stratified K-M survival analysis across different clinical features in GSE53622. Univariate (A) and multivariate (B) Cox regression analyses of the association between clinicopathological factors and overall survival (OS) of patients. (C) KM survival curves (high-risk vs low-risk patients) for overall survival in multiple subgroups.

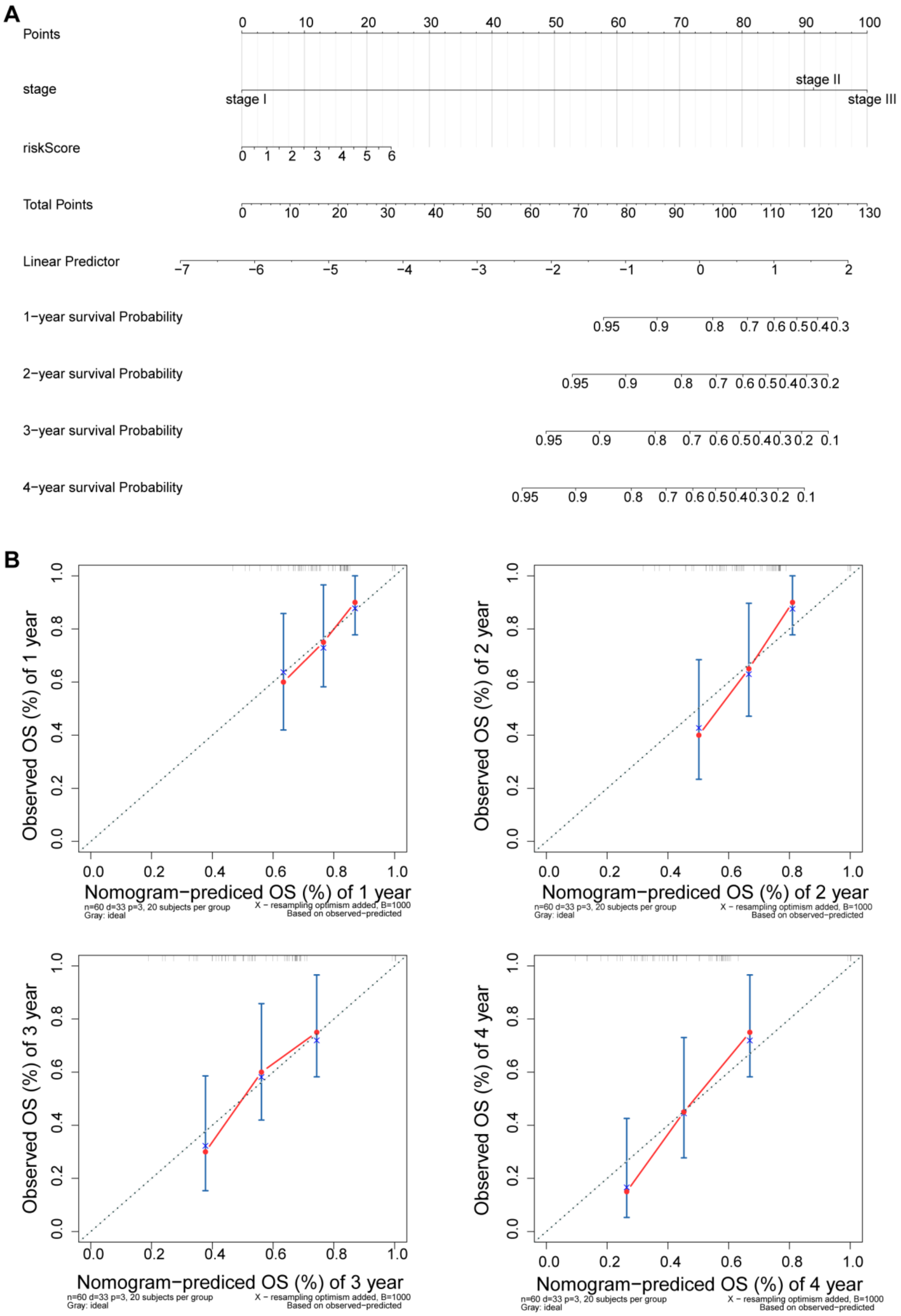

Figure S3. Construction of a nomogram for survival prediction in GSE53622. (A) Nomogram model consisting of stage and risk score factors for 1-, 2-, 3-, and 4-year OS prediction. (B) Calibration curves showing the nomogram-predicted survival probabilities corresponded closely to the actual observed proportions in 1-year prediction.

Table S1. Primer sequences for Real Time quantitative PCR.

Primers	Sequence (5' to 3')
AP000696.2 F	5'-GTTTCCCTTTTCTTCCTCCC-3'
AP000696.2 R	5'-ACTGACCTCACAGCCTCCAA-3'
LINC01711 F	5'-TGTCACAGTTTAAGGTTGGGG-3'
LINC01711 R	5'-GAGGGAGCAGTCATCGGGTAG-3'
RP11-70C1.3 F	5'-GATGCTCCTTCACCCAACT-3'
RP11-70C1.3 R	5'-TGCGTATTCAACCTCCACT-3'
AP000487.5 F	5'-GTTCCTAATCCTTCGGCTC-3'
AP000487.5 R	5'-CTTCATTCCTCTCGTGCTC-3'
AC011997.1 F	5'-AGCAATCAGCGAGACTCCGT-3'
AC011997.1 R	5'-ACCATCCATCCCTTATCCAA-3'
RP11-225N10.1 F	5'-AAGTCGTGTCTGTGGGTCTGG-3'
RP11-225N10.1 R	5'-CTTCATGCAAGGTCACTGGGT-3'
GAPDH F	5'-GGAAGGTGAAGGTCGGAGT-3'
GAPDH R	5'-TGAGGTCAATGAAGGGGTC-3'

Table S2. The results of KEGG pathway and GO function analyses on DEGs. (The details are shown in an Excel file)
